# Tumor Development in Ulcerative Colitis: Perspectives From Biomechanical Characteristics

**DOI:** 10.1111/dgd.70031

**Published:** 2025-11-20

**Authors:** Hirotaka Tao

**Affiliations:** ^1^ Department of Environmental Oncology, Institute of Industrial Ecological Sciences University of Occupational and Environmental Health, Japan Kitakyushu, Fukuoka Japan

**Keywords:** chronic inflammation, ECM mechanobiology, inflammatory immune microenvironment, tumor microenvironment, YAP

## Abstract

Throughout our lifespan, sustaining orderly morphological structures for ensuring proper functioning of organs is imperative. Among these structures, the extracellular matrix (ECM) plays a pivotal role in sustaining organ and tissue homeostasis. Nevertheless, elucidating the role of abnormal ECM‐induced biomechanical microenvironmental changes associated with transition from chronic inflammation to cancer warrants further investigation. Additionally, the temporal and spatial dynamics of the extracellular environment and immune cell populations within inflammatory regions of the body remain inadequately understood. In this review, we critically present recent analytical techniques and biomechanical approaches to elucidate how the disordered distribution of cell populations, extracellular environment heterogeneity, and changes in tissue stiffness could be interrelated in the progression from ulcerative colitis to cancer.

## 1 Introduction

It is a regrettable reality that cancer currently represents the foremost cause of mortality worldwide. The advent of an aging populace is anticipated to give rise to a marked increase in the incidence rate of cancer‐related fatalities (Sung et al. 2021). Despite the vast amount of cancer research conducted globally, a definitive solution remains elusive. This challenge is primarily due to cancer cell and tumor microenvironment (TME) heterogeneity (Greaves and Maley 2012; Quail and Joyce 2013; Black and McGranahan 2021; Chatsirisupachai et al. 2023; Swanton et al. 2024). Therefore, establishing effective tumor stratification for aiding in developing early detection and treatment guidelines is crucial, as well as strategies for effective preventive therapies, before the emergence of cancer cell diversity within the TME. To achieve this, focusing on chronic inflammation, a precancerous condition, and thoroughly understanding the physiological and pathological processes within the inflammatory immune microenvironment (IIME) at the single‐cell level are beneficial, thereby promoting more accurate tumor stratification (Grivennikov et al. 2010; Greten and Grivennikov 2019; Li et al. 2020; Hibino et al. 2021; Meli et al. 2023). In this context, active research on genetic mutations and modifications in cancer cells has garnered global attention in recent years (Borras et al. 2016; Lee‐Six et al. 2019; Hirsch et al. 2021). Nonetheless, despite the substantial accumulation of data, developing fundamental cancer therapies remains exceedingly challenging. This is because tumor formation involves genetic modifications of cancer cells with diverse characteristics during the process of cancer cell infiltration within tissues, as well as the spatiotemporally dynamic regulation of the microenvironment surrounding cancer cell populations by the extracellular matrix (ECM) (Di et al. 2023; Saraswathibhatla et al. 2023; Mierke 2024). From a biomechanical perspective, it has been hypothesized that mechanical changes occur at all hierarchical levels—cells, the ECM, and tissues—throughout all stages from chronic inflammation to cancer cell formation and metastasis (Broders‐Bondon et al. 2018; Emon et al. 2018; Nia et al. 2020; de Visser and Joyce 2023). The ECM, a biomechanical component, is an essential element of the IIME of precancerous lesions, the TME that promotes tumor formation (Sorokin 2010; Pickup et al. 2014; Marangio et al. 2022; Lo Buglio et al. 2024). It has been hypothesized that the molecular, physical, and mechanical properties of the ECM might influence the motility, survival, and function of immune cells (Campagnola 2011; Najafi et al. 2019; Sutherland et al. 2023; Mai et al. 2024). Furthermore, it has been proposed that the underlying cause of many diseases might stem from impaired communication between the ECM and immune system. Consequently, the integration of research from both the fields of ECM biology and immunology holds considerable potential for enhancing our understanding about the initiation and progression of disease and therapy. This review provides a concise synthesis of the current state of knowledge in the fields of inflammation and cancer research. It also introduces analytical techniques and approaches that have been developed to elucidate the complex relationships between cell populations, ECM heterogeneity, and changes in biomechanical tissue stiffness. Furthermore, it furnishes a synopsis of prospective trends in the domain of ECM biology.

## 2 Relationship Between Cancer Development and the Heterogeneous ECM Microenvironment

**FIGURE 1:**
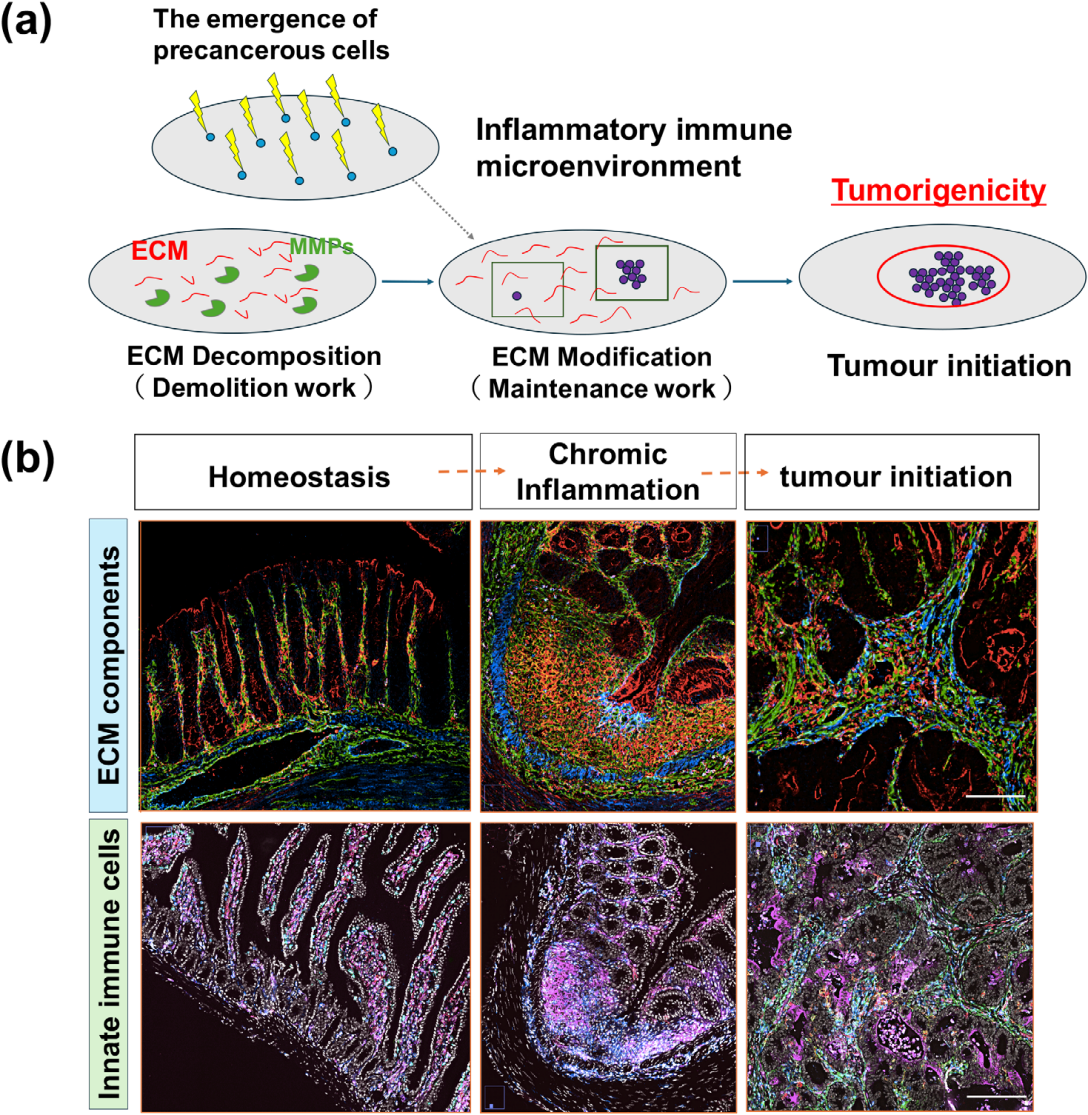
Chronic inflammation and cancer development. (a) Tumor formation in the inflammatory immune microenvironment. (b) Spatial dynamics of extracellular environment changes and immune cell populations. Scale bar = 250 mm.

Cancer is characterized by oncogenic mutations and profoundly affected by the biochemical and biomechanical characteristics of the ECM that envelops the developing tumor (Figure 1a). As the cancer advances, tumors are thought to emerge from diverse populations of cells, each possessing a variety of distinct oncogenic mutations (Visvader 2011; Greaves and Maley 2012; Black and McGranahan 2021). This cellular diversity is reflected in the tumor‐associated ECM, which shows significant variability in ECM deposition and fibrosis (Coelho and McCulloch 2016; Piersma et al. 2020; Park et al. 2023). Heterogeneity of cancer cell populations within the ECM provides insight into why therapies targeting this aspect of tumor development have not achieved notable success in clinical trials (Jin and Jin 2020). The impact of ECM heterogeneity within the IIME on tumor development and treatment efficacy remains critically unresolved.

## 3 Association Between Chronic Inflammation and Development of Colorectal Cancer (CRC)

The association between chronic inflammation and tumor initiation was initially proposed in the 19th century, based on observations that tumors frequently developed in areas of chronic inflammation and that inflammatory cells were present in tumor biopsy samples (Balkwill and Mantovani 2001). Furthermore, a substantial body of evidence, including epidemiological studies including patients and molecular biological studies using genetically engineered mice, has led to the widespread acceptance of the notion that chronic inflammation is linked to cancer (Porta et al. 2009; Grivennikov et al. 2010; Greten and Grivennikov 2019; Li et al. 2020; Hibino et al. 2021). Among these, chronic inflammation is estimated to be associated with approximately 20% of human cancers. Furthermore, there is a significant correlation between prolonged inflammation and CRC onset (Shah and Itzkowitz 2022). Inflammatory bowel disease (IBD) is characterized by a persistent inflammatory state that causes chronic mucosal damage, thereby increasing the risk of cancer development (Schmitt and Greten 2021; Park et al. 2023). The origin of inflammation in the colon is contingent upon infection and tissue damage, resulting in the recruitment of leukocytes and plasma proteins to the inflammatory site. This response primarily depends on the resident macrophages and exists in an intermediate state between basal homeostasis and classical inflammatory responses. IBD pathogenesis is complex and involves multiple factors (Zhou et al. 2019; Shah and Itzkowitz 2022; Park et al. 2023; Gudiño et al. 2025). Subsequently, the process from chronic inflammation to cancer development is mutually associated through two pathways: endogenous and exogenous (Medzhitov 2008; Visvader 2011). The endogenous pathway is activated by genetic events that initiate tumor formation. These events include mutations, chromosomal rearrangements, activation of various oncogenes through amplification, and inactivation of tumor suppressor genes. In contrast, the exogenous pathway encompasses inflammatory or infectious states associated with an increased risk of cancer development in specific anatomical locations. This pathophysiological pathway involves multiple factors, including innate immunity, adaptive immunity, gut microbiota, environmental influences, and exogenous substances (Medzhitov 2008).

## 4 Heterogeneous Microenvironment Stiffness: Effects of the ECM on Inflammation

**FIGURE 2:**
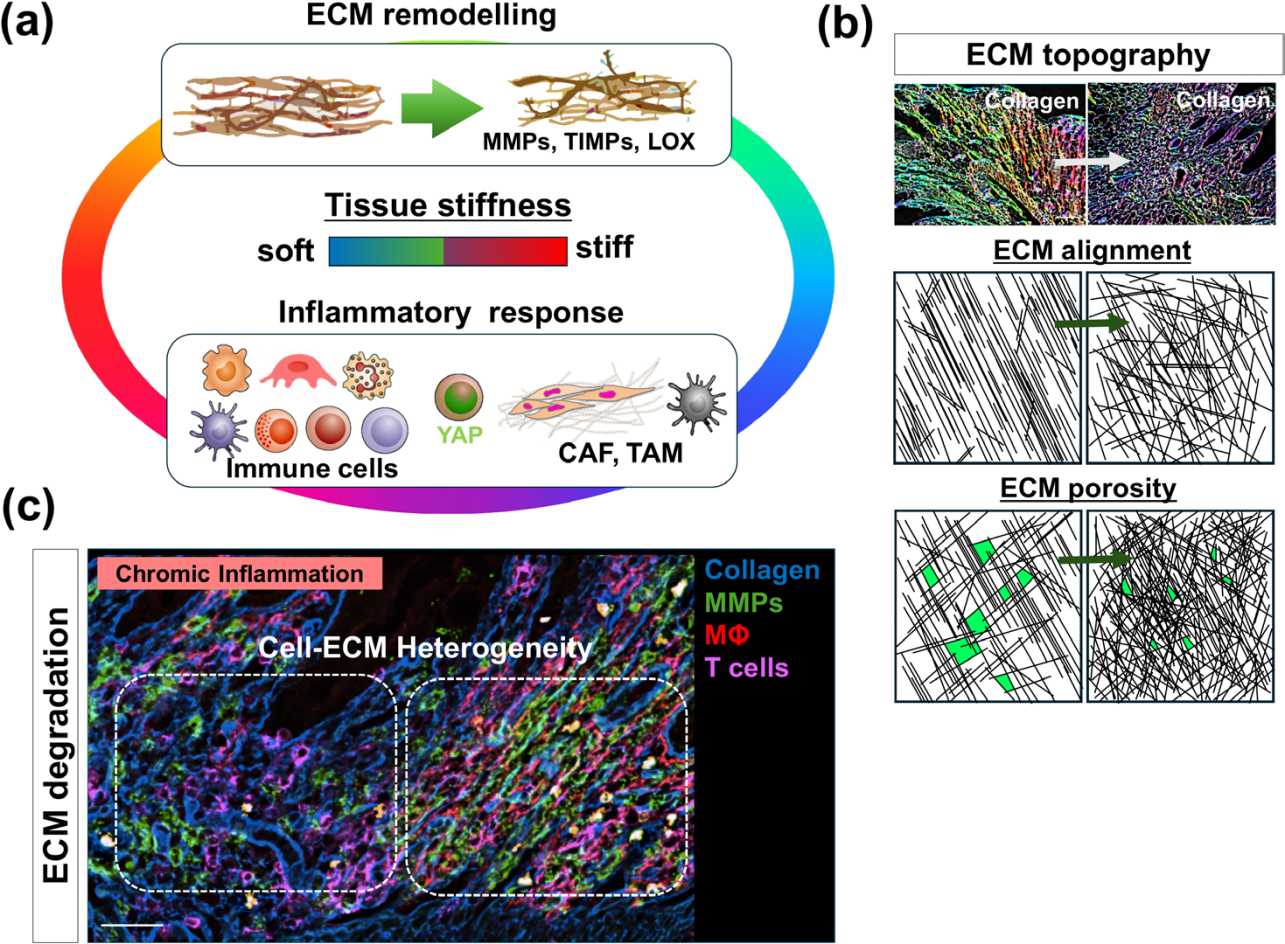
Changes in ECM degradation and in the surrounding cell populations during the chronic inflammatory phase. (a) Mechanical changes in tissue associated with ECM degradation and inflammatory responses. (b) ECM topography during chronic inflammation. Scale bar = 150 mm. (c) Cell ECM heterogeneity. Changes in MMP‐secreting immune cell populations associated with ECM degradation. Scale bar = 150 mm. ECM, extracellular matrix; MMP, matrix metalloproteinase.

The ECM serves not only as a structural scaffold but also as a biologically active component of living tissues, facilitating intercellular communication, cell proliferation, and migration (Bonnans et al. 2014; Saraswathibhatla et al. 2023; Lo Buglio et al. 2024). The ECM comprises fibrous proteins, including proteoglycans, glycosaminoglycans, different collagens (types I, III, IV, V, and VI), elastin, fibronectin, and laminin, which provide mechanical stimuli to cancer cells (Mouw et al. 2014; Saraswathibhatla et al. 2023; Lo Buglio et al. 2024). During the transition from chronic inflammation to tumor formation, the ECM and distribution of immune cell populations undergo dynamic alterations (Medzhitov 2008) (Figure 1b). Furthermore, tumor progression might be associated with increased ECM stiffness. As the ECM becomes stiffer, cancer cells might develop resistance to external forces and exhibit enhanced plasticity (Coelho and McCulloch 2016; Deng, Zhao, et al. 2022; Ishihara and Haga 2022; Fan et al. 2024). Mechanical forces within the IIME and TME might facilitate cancer cell migration through two primary mechanisms: direct and indirect transmissions via mechanosensors (Broders‐Bondon et al. 2018; Mierke 2024) (Figure 2a). Cells possess various mechanisms to perceive the biophysical characteristics of the IIME and TME and thereby mechanical forces generated within these environments (De Belly et al. 2022). These mechanosensors convert mechanical forces into biochemical signals, resulting in structural changes in the cytoskeleton of cancer cells. Additionally, the ECM serves as a source of biochemical and mechanical signals that promote tumor initiation and progression, exerting significant influence on cancer cells through bidirectional interactions mediated by the cancer cell cytoskeleton (Broders‐Bondon et al. 2018; Saraswathibhatla et al. 2023). At the cellular level, cancer cells actively respond to external forces by activating intracellular signaling pathways and binding to effectors. For instance, cell adhesion to the matrix via integrins might activate Rho GTPases and promote actin remodeling, thereby regulating cell contractility and potentially altering cell behaviors such as survival, invasion, and metastases (Piersma et al. 2020; Saraswathibhatla et al. 2023; Mierke 2024). In summary, alterations in both the tissue stiffness and inflammation, attributable to the density and composition of the ECM, including specific topography and deformability, might contribute to tumor proliferation and progression (Figure 2b). Notably, under pathological conditions that impose physical constraints, such as chronic inflammation or cancer, the mechanical properties of the ECM undergo significant changes and are implicated in the onset and progression of diseases such as IBD and CRC.

## 5 ECM Degradation in Inflammation

The precise deposition and degradation of the ECM are essential processes in the development, morphogenesis, repair, and reconstruction of biological tissues (Bonnans et al. 2014; Winkler et al. 2020; He et al. 2023) (Figure 2a). The ECM manifests in various biochemical and structural forms, with both its individual components and its three‐dimensional ultrastructure transmitting specific signals to cells, thereby regulating critical functions in the early stages of inflammation, such as the migration and differentiation of immune cells (Sorokin 2010). Under physiological conditions, ECM degradation and remodeling are meticulously regulated by a large family of enzyme proteins, including matrix metalloproteinases (MMPs) (Bonnans et al. 2014), the tissue inhibitors of metalloproteinases (TIMPs) (Cabral‐Pacheco et al. 2020), crosslinking transglutaminase, and lysine oxidase (LOX), among others (Vallet and Ricard‐Blum 2019). These enzymes are thought to comprehensively degrade various ECM proteins and modulate pathways and interactions such as cell‐matrix interactions, cell–cell interactions, and growth factor secretion, through the cleavage of cell‐surface receptors (Bonnans et al. 2014; Cabral‐Pacheco et al. 2020). Recently, a biomechanical perspective on how ECM forces regulate immune cell behavior in inflammatory tissues has garnered attention (Du et al. 2022). Notably, MMPs, which are responsible for collagen degradation, have been reported to actively contribute to immune responses by inducing abnormal ECM expression and tissue remodeling processes (Bonnans et al. 2014). Furthermore, MMP9 has been implicated in promoting tumor cell infiltration and metastasis by degrading type IV collagen and degraded collagen molecules (Kessenbrock et al. 2010; Huang 2018; Mondal et al. 2020). In chronically inflamed tissues, inflammatory‐associated cells contribute to dynamic changes in the ECM through MMP9 expression in collaboration with macrophages (Figure 2c). Additionally, ECM changes mediated by MMP9 might induce significant structural alterations in the inflamed stroma, potentially leading to cancer development and tissue fibrosis. This fibrotic stroma is reported to be present in over 50% of patients with CRCs. Additionally, the activity of MMP9 in stroma remodeling suggests an association with more aggressive tumor phenotypes (Bonnans et al. 2014).

## 6 Relationship Between ECM and the Immune System

The immune response is intricately regulated by signals from the tissue microenvironment, encompassing both biochemical and mechanical cues. These signals, originating from the tissues, ECM, and their constituent cells, are crucial for modulating immune cell functions (Du et al. 2022; Mai et al. 2024). Within an inflammatory setting, a diverse array of inflammatory cells, such as macrophages, granulocytes, monocytes, and mast cells, infiltrate the IIME. These cells interact with tumors through cytokine secretion, creating a complex IIME that exhibits immunosuppressive properties, encourages dedifferentiation of mutated cells, and influences tumor biology. The IIME's complexity is highlighted by the variety of infiltrating inflammatory cells, secretion of inflammatory mediators, and interactions among components such as hypoxia, acidity, and low glucose levels, alongside the plasticity of inflammatory cell infiltration and differentiation. Beyond inflammatory cells, the IIME includes innate immune cells (natural killer cells), adaptive immune cells (Tregs, B cells), cancer cells, cancer‐associated fibroblasts (CAFs), endothelial cells, and the surrounding ECM. These entities interact through direct or indirect contact, cytokine and chemokine secretion, and autocrine or paracrine pathways, thereby regulating and promoting tumor proliferation (Quail and Joyce 2013; Li et al. 2020; de Visser and Joyce 2023). It is suggested that changes in the biophysical properties of tissues in various disease states, inflammatory conditions, and aging may alter the mechanical signals inherent to cells (Broders‐Bondon et al. 2018; Du et al. 2022; Di et al. 2023). These mechanical signals are converted into biochemical signals through mechanotransduction processes, with several mechanotransduction pathways identified in immune cells (Du et al. 2022; Mai et al. 2024; Wang et al. 2024). These pathways are thought to influence essential cellular functions, in addition to metabolism and cell migration (Vasudevan et al. 2023; Mierke 2024; Wang et al. 2024). The potential role of alterations in tissue stiffness as a novel “danger signal” which might alert both the innate and adaptive immune systems to the presence of injury or infection should be considered.

## 7 Relationship Between ECM and the Tumor Stroma

Tissue mechanics undergo temporal modifications during infection and inflammatory responses, adding a dynamic layer to immune regulation. There is a growing interest in understanding how mechanical signals within the tissue environment could influence immune cell behavior and initiate, propagate, and resolve immune responses. The tumor stroma is characterized by an abnormal ECM with unique mechanical properties and various cell types, including CAFs and immune cells, playing a pivotal role (Kobayashi et al. 2019; Sahai et al. 2020; Barbazan et al. 2023). The stroma engages in multiple bidirectional interactions with tumor cells, establishing interdependent relationships that are essential for tumor formation. CAFs are identified as the primary drivers of ECM stiffening and degradation (Kobayashi et al. 2019; Sahai et al. 2020; Yang et al. 2023; Lan et al. 2025; Liu et al. 2025). CAFs interact with nearly all cells within the IIME and TME, and possess the ability to regulate ECM components crucial for tumor formation (Kobayashi et al. 2019; Sahai et al. 2020; Yang et al. 2023; Lan et al. 2025; Liu et al. 2025). Interactions between CAFs, cancer cells, and M2 macrophages are critically important for ECM stiffening and degradation (Mao et al. 2021). CAFs induce hypoxia within the TME, which is a key factor in inducing both stiffness and degradation. The regulatory function of integrin receptors in cancer cells is crucial for modulating the fate of ECM components. These processes—cancer cell proliferation, migration, and invasion—are outcomes of ECM stiffness and degradation. Therefore, maintaining ECM homeostasis to enhance drug penetration and improve the efficacy of antitumor strategies is a promising approach. However, the response of immune cells to the heterogeneous mechanical microenvironment of tumors has been insufficiently studied. Further research is necessary to elucidate the extent to which therapeutic modification of the ECM could influence tumor stalling and associated immune responses, thereby improving clinical outcomes.

## 8 ECM Deposition and Crosslinking During Fibrosis

The ECM is a complex network of fibers that provides structural support to tissues and serves as a medium transmitting biophysical and biochemical signals to cells. Cells actively remodel the ECM through dynamic processes such as matrix degradation and synthesis and mechanical remodeling, facilitating intercellular communication (Theocharis et al. 2019; Lloyd and He 2024; Mayorca‐Guiliani et al. 2025). The mechanical properties of ECM‐containing cells differ fundamentally from those of ECM without cells, underscoring their crucial role in active remodeling that imparts physiological tissue characteristics (Du et al. 2022; Mai et al. 2024). The signals provided by the ECM regulate numerous cellular functions involved in maintaining homeostasis and disease progression (Broders‐Bondon et al. 2018; Blanco et al. 2023; Xin et al. 2023). Fibrosis results from abnormal wound‐healing processes driven by chronic inflammation or continuous injury, characterized by excessive accumulation of ECM around inflamed or injured tissues, leading to impaired physiological functions and potential organ failure. Fibrosis can affect multiple organs, including the liver, kidneys, heart, lungs, skin, and intestines, and can be induced by various diseases such as cirrhosis, chronic kidney disease, heart failure, idiopathic pulmonary fibrosis, scleroderma, and IBD (Mayorca‐Guiliani et al. 2025). In cases of fibrosis, activated myofibroblasts produce excessive fibrogenic proteins (Saraswathibhatla et al. 2023). These proteins comprise fibrogenic collagen types I, III, IV, and V, fibronectin, and elastin (Theocharis et al. 2019). The accumulation of these proteins is balanced by ECM remodeling enzymes, such as MMPs, adamalysin, or meprin (Lu et al. 2011; Bonnans et al. 2014; Theocharis et al. 2019; Zhao et al. 2022). The enzymatic activity of MMPs and adamalysin is regulated by the TIMP family proteins, which prevent excessive ECM degradation (Cabral‐Pacheco et al. 2020). The imbalance between ECM accumulation and remodeling is a core characteristic of fibrosis. Enzymatic crosslinking is mediated by multiple enzyme groups including LOX and transglutaminase proteins (Vallet and Ricard‐Blum 2019; Lloyd and He 2024). Although lysine hydroxylase enzymes do not directly contribute to crosslinking reactions, they play a crucial role in LOX‐mediated crosslinking (Lloyd and He 2024). In addition to the regulation by degradative enzymes, ECM proteins undergo post‐translational modifications that alter their structure and function (Lloyd and He 2024). Over‐crosslinked and hardened ECM is a characteristic feature of fibrotic diseases in various organs, such as the lungs, liver, and skin (Mayorca‐Guiliani et al. 2025). It is also an important pathological driver of progressive fibrosis (Theocharis et al. 2019; Mak 2020). This presents a unique opportunity for developing future therapeutic strategies focused on targeting the ECM in the context of fibrosis.

## 9 Relationship of YAP/TAZ from Chronic Inflammation to Tumor Development

YAP/TAZ function as transcriptional co‐activators that are frequently upregulated in human solid tumors; their roles within cancer cells are currently the subject of extensive research (Zanconato et al. 2019; Liang et al. 2022; Piccolo et al. 2023; Zhao et al. 2023). Numerous studies have established that YAP/TAZ are essential for initiating various cellular autonomous responses, including sustained proliferation, cellular plasticity, treatment resistance, and metastasis (Zanconato et al. 2019; Cai et al. 2021; Piccolo et al. 2023). However, tumors are complex entities, and cancer cells represent only one component of intricate tumor tissues. Moreover, the mechanistic regulatory pathways of YAP/TAZ are highly context‐dependent, as evidenced in the existing literature (Zanconato et al. 2019; Cai et al. 2021; Piccolo et al. 2023). Dupont et al. demonstrated that YAP/TAZ activity is directly modulated by mechanical and physical stimuli originating from the extracellular environment, specifically ECM stiffness and cell geometry (Dupont et al. 2011). This finding reveals a novel dimension of YAP/TAZ regulation that extends beyond traditional biochemical signaling pathways. Incorporating mechanical and physical factors into YAP/TAZ regulation fundamentally alters our understanding of cell signaling in several aspects. This study illustrates that cell signaling is not only confined to biochemical pathways but also includes mechanical and physical factors mediated by ECM stiffness. Consequently, the concept of intercellular communication has been broadened to encompass a more comprehensive framework that recognizes force and structural changes as significant signaling mechanisms. Focusing on chronic inflammation in colon tissue, research has also elucidated the role of YAP/TAZ in inflammation and immunity (Yui et al. 2018; Zhou et al. 2019; Pan et al. 2019; Chen, Jin, et al. 2023; Meli et al. 2023; Wang et al. 2025). In mouse models of IBD, YAP expression regulates the crosstalk between immune and tumor cells within the IIME, affecting T cells, bone marrow–derived suppressor cells, and macrophages. Furthermore, YAP expression is subject to distinct regulatory mechanisms during the induction of M2 and M1 macrophages (Zhou et al. 2019; Wang et al. 2025). These findings suggest that a comprehensive understanding of the multifunctionality of YAP in different cell types is critically important for the therapeutic management of IBD. Of note, Yui et al. demonstrated the essential function of ECM remodeling for activating YAP/TAZ during tissue repair using an IBD mouse model (Yui et al. 2018). This process involves multiple interrelated mechanisms in the context of inflammatory damage and wound healing. During tissue repair, colonic epithelial tissue undergoes significant reprogramming, reverting to a primitive state akin to the fetal stage. This reprogramming is orchestrated by ECM remodeling, FAK/Src signaling pathway activation, and subsequent YAP/TAZ activation. Repaired epithelium, characterized by Sca1 expression, displays distinct cellular and molecular profiles compared with the normal epithelium. YAP/TAZ is a crucial mechanosensor for tissue reprogramming and is essential for effective tissue repair. The authors developed an in vitro model using type I collagen matrix and Wnt ligands, which successfully replicated the reprogramming observed in vivo. This model sustains endogenous YAP/TAZ activation and induces cell‐fate conversion. Reprogramming into a fetal‐like state is reversible both in vitro and in vivo. Following complete regeneration, tissue restores its normal cellular structure and adult‐specific gene expression. YAP/TAZ‐mediated activation is both necessary and sufficient to establish a repair epithelial‐like state in vitro. This study offers insights into elucidating the mechanisms underlying tissue repair and proposes potential therapeutic strategies for enhancing regeneration in patients with IBD. In recent years, diagnostic technologies employing organoids for patients with IBD and CRC have been rapidly developed (Dotti and Salas 2018; Wakisaka et al. 2022; Sakshaug et al. 2023; Heydari et al. 2025; Wu et al. 2025). Concurrently, applications of organoid technology that model high‐YAP and fetal‐like epithelial states are emerging as potent tools for elucidating the underlying mechanisms (Chen, Qiu, et al. 2023; Kobayashi et al. 2022).

## 10 YAP‐Containing Mehcanotransduction During Tumor Development

YAP response to mechanical stimuli manifests significant variation between normal and diseased tissues, primarily due to alterations in the microenvironment mechanical properties in diseased tissues and changes in cellular mechanosensitivity (Zanconato et al. 2019; Piccolo et al. 2023). Mechanisms underlying the YAP‐containing mechanotransduction pathway might enhance our understanding of disease processes such as cancer and fibrosis from multiple perspectives (Panciera et al. 2017; Cai et al. 2021; He et al. 2022). In normal tissues, YAP activity is tightly regulated and activated in regions where cell proliferation and stem cell maintenance are crucial, such as the basal layer of the skin or base of the intestinal crypts (Deng, Wu, et al. 2022). Under normal conditions, YAP activation is transient and context‐dependent, which facilitates appropriate tissue homeostasis and regeneration (Deng, Zhao, et al. 2022; Fu et al. 2022). Conversely, under pathological states, abnormal mechanical properties of tissues often result in sustained and aberrant activation of YAP. In cases of diseases, extensive ECM remodeling that alters cell–ECM interactions is frequently observed. For instance, in patients with tumors, increased ECM stiffness triggers sustained YAP activation in cancer cells and CAFs (Athavale et al. 2024). Similarly, in liver fibrosis cases, activation of hepatic stellate cells leads to increased collagen deposition and ECM stiffness, further activating YAP (Guo et al. 2023). This creates a positive feedback loop, where YAP activation further promotes ECM production and ECM stiffness, exacerbating the disease state. These findings suggest that changes in the ECM composition and mechanical properties play a role in cancer progression and metastasis. Understanding how cancer cells respond to these physical stimuli via the YAP signaling pathway might elucidate the mechanisms underlying tumor growth and spread. Furthermore, YAP activity has been reported to influence stem cell differentiation in response to mechanical stimuli (LeBlanc et al. 2021; Deng, Wu, et al. 2022). This might provide insight into interpreting how mechanical changes in tissues during disease could affect stem cell behavior and tissue regeneration. This suggests that tissue homeostasis depends on the balance between growth factor signaling and mechanical stimuli. Disruption in this balance might explain the mechanism through which mechanical changes in diseased tissues promote pathological progression. This understanding opens new avenues for disease research and therapeutic approaches that integrate the biochemical and mechanical aspects of the cellular microenvironment in diseases, such as cancer and fibrosis.

## 11 Spatial Stiffness Mapping Technology in Cancer Development

Tissue mechanics represents a fundamental physical property that emerges during the formation, development, and aging of biological systems (Chaudhuri et al. 2020; Lin et al. 2024; Phillip et al. 2015; Saraswathibhatla et al. 2023; Mierke 2024). Gaining insights into tissue mechanics is a promising approach to unraveling the fundamental mechanisms that drive chronic inflammation and tumor formation. These conditions are complex, involving multifaceted factors such as alterations in cancer cells, tissues, and organs, as well as signals from the surrounding microenvironment. Consequently, a comprehensive approach is essential for effective analysis. In recent years, although a variety of techniques have been developed to investigate tumor mechanics, challenges in measurement accuracy persist. Among these techniques, atomic force microscopy (AFM) has proven to be a highly effective tool for concurrently assessing the structural and mechanical properties of biological materials, ranging between individual intracellular molecules and extracellular structures and entire tissue samples, with exceptional spatiotemporal resolution. This advancement has expanded the possibilities for understanding the physical characteristics of tumors and significantly contributed to cancer research (Dufrêne et al. 2017; Li, Liu, et al. 2021; Li, Xi, et al. 2021). AFM is also actively used in clinical examinations of both healthy and malignant tissues, particularly in the context of chronic inflammation in the colon tissue (Tian et al. 2023). To date, several techniques have been developed to measure the mechanical properties of soft biological samples, including AFM (Dufrêne et al. 2017; Krieg and Muller 2019; Calò et al. 2020; Horikiri et al. 2024), magnetic tweezers (Zhu, Tao, et al. 2020; Zhu, Zhang, et al. 2020; Wang et al. 2020; Choi et al. 2022), shear rheometers (Deptuła et al. 2020), tactile sensors (Lee and Ahn 2020; Huang et al. 2025), ultrasound elastography (Nia et al. 2016; Sigrist et al. 2017; Kumar et al. 2023; Zhu et al. 2025), and micropillars (De Saram et al. 2024). The physical quantities obtained through these techniques should be assessed in comparison with other measurement approaches. Considering the dimensions of the tissue and whether the area of interest is superficial or deep when determining suitable measurement methods are essential. Additionally, a comprehensive discussion about the implications of selecting between invasive and noninvasive measurement techniques is warranted. The following is a brief introduction to the current advantages and limitations of the representative mechanical measurement techniques. Considering AFM, it offers nanoscale resolution, facilitating detailed high‐resolution measurements of the mechanical properties of individual cells and intracellular structures (Krieg and Muller 2019). AFM quantitatively assesses the Young's modulus, adhesion forces, and other mechanical characteristics. It is applicable to diverse arrays of materials, including biological tissues, cells, and individual molecules. Additionally, by functionalizing the AFM probe with specific molecules, analyzing specific interactions between cells and the extracellular matrix is possible. However, AFM's limited scanning range poses challenges for investigating extensive tissue areas. Additionally, the requirement for physical contact with the sample might result in tissue damage or alterations. Regarding ultrasound elastography, it, along with other elastography techniques such as magnetic resonance elastography (MRE), enables the imaging of tissue stiffness within the body (Ce et al. 2023; Oglat and Abukhalil, 2024). These techniques can be used to map stiffness in extensive tissue areas and offer a comprehensive perspective on tissue mechanics. Ultrasound elastography is generally noninvasive and can be used to evaluate tissue stiffness; however, its accuracy might decrease in deep tissue regions owing to vibration energy attenuation. The anisotropic nature of muscle‐like tissues, where mechanical properties vary with the measurement direction, might also affect the accuracy of elastography. In regard to magnetic tweezers, they measure forces at the piconewton (10^−12^ N) scale, facilitating the study of mechanical properties of interactions between small molecules and biomolecules. They allow real‐time observation of molecular behavior under controlled forces, providing insights into understanding dynamic processes such as DNA stretching, protein folding, and motor protein activity. Magnetic tweezers are applicable to a variety of biomolecules including DNA, RNA, proteins, and polysaccharides (Choi et al. 2022). However, the magnetic beads used in magnetic tweezers might interact with the target molecules, potentially introducing artifacts into the measurements. With respect to micropillars, their use for mechanical measurements constitutes a technique that directly quantifies the forces acting on pillars as a result of cell adhesion or movement. This method offers the advantage of determining both the magnitude and direction of the forces exerted by cells with high spatial resolution. Consequently, this enables the evaluation of forces at specific cell adhesion points or active regions, thereby facilitating a comprehensive understanding about cellular activity. The simultaneous measurement of the direction and magnitude of the forces exerted by the cells allows for a detailed analysis of the complex mechanical behavior (De Saram et al. 2024). In addition, micropillars might physically stimulate cells or influence cell proliferation and movement. Regarding flexible tactile sensors, they present significant advantages in artificial tactile systems, including adaptability to complex surfaces, enhanced sensitivity, and the ability to mimic human‐like tactile perceptions. These sensors can detect a wide range of physical signals such as pressure, temperature, and vibration (Huang et al. 2025). This functionality enables accurate identification of surface properties and rapid adaptation to environmental changes. However, measurements in deep tissue regions remain a considerable challenge. Considering shear rheometer, it applies an external force (stress) to a material and measures its response (strain or flow) to evaluate rheological properties, such as viscosity and elasticity. In medical settings, it is used for measuring the viscoelasticity and fluidity of blood and bodily fluids (e.g., saliva and mucus) to assist in disease diagnosis and progression assessment. The limitations of this technique include issues related to sample preparation, necessity for precise measurement of shear rate and stress, and the potential for measurement artifacts. In biological tissues characterized by spatially and temporally regulated mechanical properties, integrating conventional mechanical property assessment techniques with more precise and localized evaluations would enhance our understanding about the variability in intrinsic mechanical properties and intercellular interactions at both the cellular and organ levels.

## 12 Multi‐Omics Technologies in Cancer Research

Spatiotemporally integrated single‐cell multi‐omics technologies and methodologies are advancing through technological innovation and applied data processing (Carranza et al. 2024; Bressan et al. 2023; Vandereyken et al. 2023; Baysoy et al. 2023; Ning et al. 2023). By concurrently integrating various single‐modality omics approaches, these technologies offer a highly effective means of characterizing cellular states and activities during chronic inflammation and carcinogenic processes (Fiocchi 2023). Below, we introduce some of these technologies.

### 12.1 Single‐Cell Sequencing Technology

**FIGURE 3:**
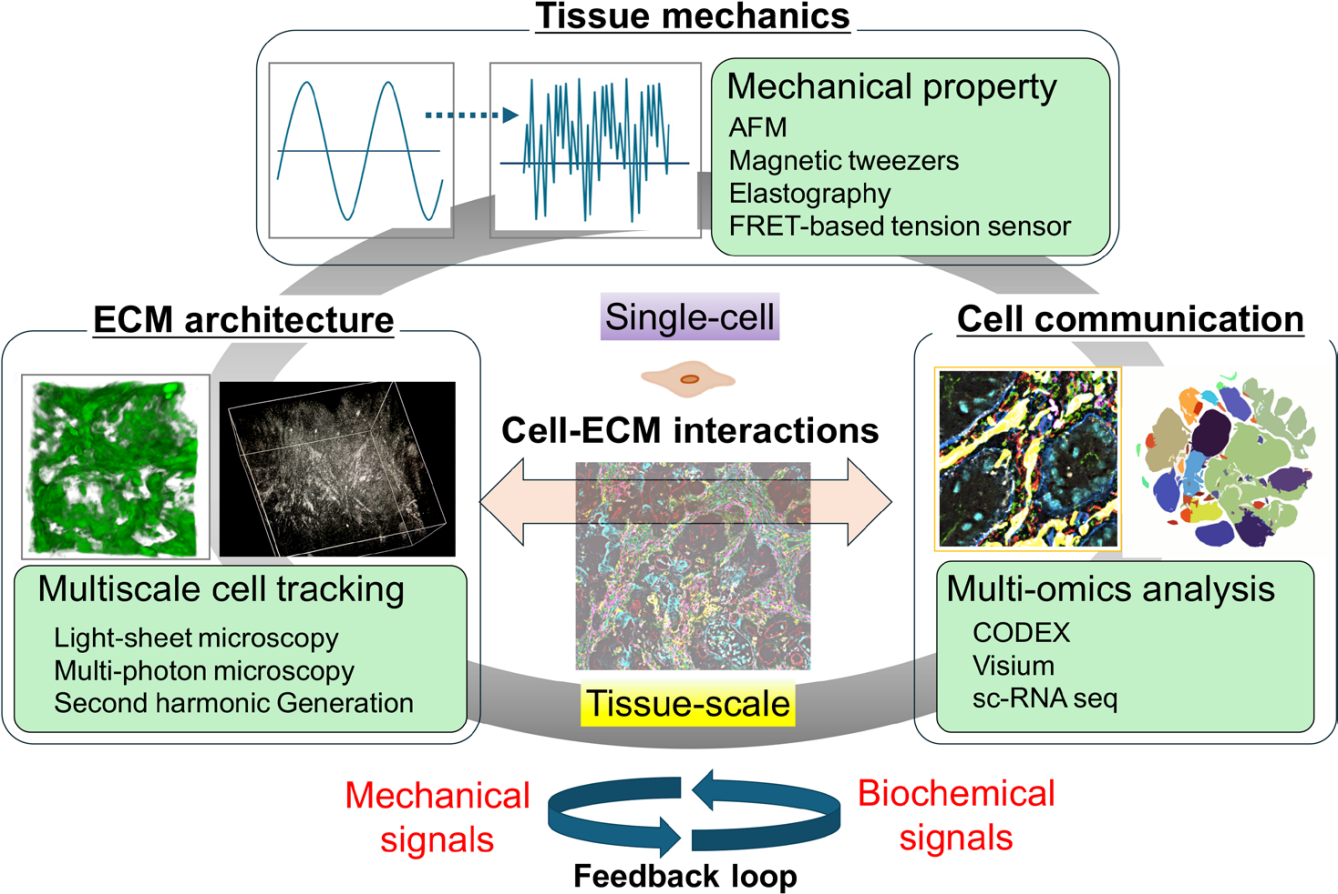
Integrated multiple approaches to comprehensively understand biophysical and pathological processes in the IIME at multicellular scales. IIME, inflammatory immune microenvironment.

Single‐cell RNA sequencing (scRNA‐seq) facilitates the examination of transcriptomes at the single‐cell level within complex tissues, with large‐scale analyses advancing globally (Marx 2021; Zhao et al. 2023). This technology allows the high‐throughput analysis of gene expression variations influenced by genomic and epigenetic factors, as well as the identification of novel cell‐specific markers and cell types. It continues to yield significant insights into the immune microenvironment of tumors, detection of tumor heterogeneity, and exploration of metastasis mechanisms (Rao et al. 2021; Zhao et al. 2023; Gudiño et al. 2025). scRNA‐seq elucidates tumor heterogeneity, monitors tumor progression (Bindea et al. 2013), and facilitates classification of immune cells, identification of immune cell evasion mechanisms, drug resistance mechanisms, and development of effective clinical target therapies in conjunction with immunotherapy. The expression of various immune mediators and regulatory factors within the IIME, along with the abundance and activation state of various cell types, determines whether immune balance and inflammation promote or suppress tumor proliferation (Boland et al. 2020; Moncada et al. 2020). Consequently, elucidating the characteristics of inflammatory infiltrating cells is critically important for understanding the roles of immune mediators and regulatory factors in the IIME (Smillie et al. 2019; Korsunsky et al. 2022; Oliver et al. 2024) (Figure 3).

### 12.2 Analysis of the Spatiotemporal Dynamics of Tumor‐Infiltrating Immune Cells Using the CODEX

IIME and TME encompass a diverse array of intracellular and extracellular components that promote tumor proliferation, invasion, and metastasis (de Visser and Joyce 2023). Solid tumors are distinguished by a variety of cell types organized in spatially structured configurations, exhibiting considerable heterogeneity both intra‐ and inter‐tumorally (Bindea et al. 2013). Over the past decade, advancements in spatial profiling technologies have emerged, offering the potential for determining the complexity of these cellular structures and enhancing our understanding about the intricate molecular mechanisms underlying tumor ecology (Elhanani et al. 2023). In 2018, a research team led by Garry Nolan introduced the CODEX multiplex immunofluorescence imaging technology (Goltsev et al. 2018; Schürch et al. 2020; Black et al. 2021; Kuswanto et al. 2023; Mayer et al. 2023). These biomarker panels can be customized to elucidate cell types and their functional states. Spatial interactions between these markers can aid in identifying cell–cell interactions and cell neighborhoods. Imaging technologies such as the CODEX, which spatially map the state of immune cells within tissues at single‐cell resolution, are transforming biomarker‐based diagnostics and providing novel therapeutic insights across various disease areas (Elhanani et al. 2023). The advanced multiplex tissue visualization technology and single‐cell spatial resolution of the CODEX are anticipated to facilitate the analysis of spatial relationships between different cells in the future, offering significant insights into the fundamental structural pathophysiology of chronic inflammation and cancer. The advancement in spatial transcriptome analysis technology—specifically Visium—has facilitated the examination of gene expression while maintaining the spatial context of cells (Ståhl et al. 2016; Salmén et al. 2018). Recently, Visium spatial gene analysis has been integrated with the CODEX, addressing each other's technical limitations and advancing analysis from two‐dimensional to three‐dimensional tissue analysis (Elhanani et al. 2023; Mo et al. 2024). The spatial relationships between IIME and TME components have been elucidated, providing a systematic perspective. The number of single‐cell analysis technologies incorporating spatial resolution continues to expand (Bandyopadhyay et al. 2024). This integrated analytical approach is expected to enable the identification of characteristics of immune cell populations and the spatiotemporal dynamics of the ECM environment within the IIME during progression from chronic inflammation to cancer, thereby elucidating environmental factors contributing to tumor formation (Figure 3).

## 13 Future Prospects in Biomechanical Analysis of the Inflammatory/Immune Microenvironment

Recognizing the significance of the IIME and TME has prompted a paradigm shift in cancer research, transitioning from a tumor‐centric approach to one that encompasses the entire IIME and TME. The stiffening and remodeling of the ECM are interrelated processes that form a vicious cycle, promoting cancer progression. As ECM stiffening advances, mechanical signal transduction is activated, resulting in the secretion of MMPs from both cancer and stromal cells. Increased MMP activity has been demonstrated to facilitate the degradation and reorganization of ECM components. Consequently, ECM stiffening is a highly dynamic process, extending from chronic inflammation to the early stages of cancer. ECM remodeling associated with mechanical transmission is critically important for activating cancer‐associated stromal cells, tumor angiogenesis, immune evasion, and tumor cell migration and invasion. Mechanical signals associated with ECM stiffness have been shown to influence the contractility of the cytoskeleton of tumor and stromal cells. The dynamics of integrins and focal adhesions are significant mediators of cancer progression induced by ECM hardening. However, other factors involved in the response to ECM stiffening and those promoting mechanical signal transduction to all components within tumor remain to be identified. Further research is necessary to elucidate the mechanisms by which ECM stiffening regulates the IIME and TME, immune surveillance, and cancer metastasis. Changes in the mechanical properties of tissues are common in pathological conditions such as aging, tissue injury, inflammation, and tumor formation. These mechanical modifications evidently have the capacity to influence immune cells, and thereby impact their effector functions (Marino and Weeraratna, 2020; Statzer et al. 2023). Changes in ECM, the underlying tissue remodeling, and mechanical transmission pathways that detect tissue mechanics are potential therapeutic targets for diseases such as fibrosis and cancer (Theocharis et al. 2019; Di et al. 2023; Lloyd and He 2024; Mayorca‐Guiliani et al. 2025). Recent innovations in microscopy techniques (Poole and Mostaço‐Guidolin 2021), second‐harmonic generation methods (Campagnola 2011; Chen et al. 2012; Pichon et al. 2022), and also the spectacular mechanical measurement method (Narasimhan et al. 2020; Molnar and Manneville 2025) have been developed alongside live imaging labeling methods for ECM (Isser et al. 2023; Fiore et al. 2025) to analyze the three‐dimensional dynamics of the ECM. Based on the insights and technological advancements discussed in this review, further improvements in the methods for measuring tissue mechanics in vivo and integrated analyses are urgently needed. By further investigating the regulation of immune responses through tissue mechanics, new insights, which could lead to the alleviation or resolution of inflammation, treatment of autoimmune diseases, and promotion of regenerative medicine, are undoubtedly within reach.

## Conflicts of Interest

The author declares no conflicts of interest.

## Data Availability

The data that support the findings of this study are available from the corresponding author upon reasonable request.
